# The Anti-CD38 Antibody Therapy in Multiple Myeloma

**DOI:** 10.3390/cells8121629

**Published:** 2019-12-12

**Authors:** Maria Teresa Petrucci, Federico Vozella

**Affiliations:** Hematology, Department of Translational and Precision Medicine, Azienda Ospedaliera Policlinico Umberto I, Sapienza University of Rome, Via Benevento 6, 00161 Rome, Italy; federico.vozella@gmail.com

**Keywords:** multiple myeloma, monoclonal antibodies (MoAb), CD38+

## Abstract

Multiple myeloma (MM) is the second-most common hematologic malignancy after diffuse large B-cell lymphoma. Despite the improvement in response and survival rates following the introduction of novel therapies, only a few patients are cured, and the majority of MM patients experience several relapses and receive multiple lines of treatment. Currently, bortezomib and lenalidomide are the core component of treatment both at the time of diagnosis and at the relapse as well as the new proteasome inhibitors (PIs), such as carfilzomib and ixazomib, and the next-generation immunomodulatory drug, pomalidomide, are now available for patients in relapse. In addition, drugs with a different mechanism of action, such as the histone deacetylase inhibitor and the monoclonal antibodies (MoAb) targeting SLAMF7 or CD38, are a part of the anti-myeloma armamentarium and are very important for heavily pretreated or double refractory to a PI and IMiD patients. In this paper, we focus on the efficacy as well as toxicities of CD38 antibodies used both as a single agent and in combination as multiple myeloma treatment.

## 1. Daratumumab in Monotherapy for Relapse/Refractory Patients

Daratumumab is a first-in-class human monoclonal antibody that targets CD38, approved in the US by the Food and Drug Administration (FDA) and in Europe by the European Medicines Agency (EMA) in 2015 and 2016, respectively. It was permitted for the treatment of multiple myeloma (MM) patients who have received at least three prior lines of therapy, including a proteasome inhibitor (PI) and an IMiD, or who are double refractory to a PI and an IMiD. The initial approval of this anti CD38, as a monotherapy, was supported by evidence from two key clinical trials: one pivotal Phase II trial, MMY2002 [1], and one supportive Phase I/II trial, GEN501 [2]. Both of these studies were conducted in heavily pretreated patients with MM or those refractory to a PI and an IMiD. Collectively, the results from these studies indicate that daratumumab is a safe and efficacious treatment that provides clinically meaningful and durable response rates, depth of response, progression-free survival (PFS), and overall survival (OS) [3]. 

## 2. Pivotal Phase II Study MMY2002

MMY2002 was an open-label study, single-agent, multicenter, two-part study of daratumumab monotherapy conducted in 124 subjects with heavily pretreated or highly refractory MM. Among the 124 patients, 106 were treated in the 16-mg/kg group. At the end of Part 1 Stage 1, the 8-mg/kg dose was discontinued after the treatment of 18 patients, as the observed overall response rate (ORR) did not meet the protocol-specified criteria for continuation. Three subjects in the 8-mg/kg group crossed over to the 16-mg/kg group but remained included as part of the 8-mg/kg group analyses. Based on the cumulative response data, the 16-mg/kg group was further expanded in Part 2. Subjects had received a median of five prior lines of therapy, with 95% refractory to a PI and an IMiD, 63% refractory to pomalidomide, and 48% refractory to carfilzomib. The primary efficacy endpoint was the ORR, defined as the percentage of subjects who achieved a partial response (PR) or better. Key secondary endpoints included the duration of response, time to response, time to disease progression, PFS, and OS. Daratumumab 16 mg/kg demonstrated remarkable clinical activity in patients with MM that were heavily pretreated or highly refractory, providing an ORR of 29%. The response to treatment was rapid and durable, with a median time to response of 1 month and a median duration of response of 7.4 months. Daratumumab additionally demonstrated a compelling depth of response (very good PR [VGPR] or better rate of 12%), as indicated by three stringent complete response (sCRs) and 10 VGPRs. This outcome is noteworthy, and the depth of response to daratumumab was also observed to improve with continued treatment. The three subjects who achieved a sCR remained in complete remission at the time of clinical cut-off, with follow-up durations ranging from 9 to 14 months. This indicates that these subjects not only had a deep response but that their response was durable. Importantly, evidence indicates that deeper responses are associated with improved survival [4]. The response to daratumumab monotherapy was consistent across all subgroups, regardless of the number of prior lines of therapy, refractory status, or geographic region. Of particular importance, this response was independent of the prior therapy received. That is, a substantial number of subjects in Study MMY2002 were already refractory to pomalidomide and carfilzomib; these individuals had ORRs (28% and 29%, respectively) and depth of responses (VGPR or better rates of 13% and 12%, respectively) that were similar to those in the overall population. As a result of the observed depth of response, treatment with daratumumab induced complete or near-complete bone marrow clearance of the malignant plasma cell clone in some responders. Among 12 subjects with baseline bone marrow plasma cell involvement greater than 30%, five subjects (42%) had post-baseline plasma cell involvement normalized to <5%; an additional four subjects (33%) had post-baseline plasma cell involvement improved from 5% to 10%. Daratumumab also showed compelling survival results. At the time of the primary analysis, after approximately nine months of follow-up, median PFS was 3.7 months and median OS had not yet been reached. The 12-month OS rate was 65% (95% CI: 51–76%). Ninety-seven percent of responders were still alive compared with 60% of non-responders. In responders to treatment, 6- and 12-month OS rates were 100% and 96%, respectively. In an updated analysis, OS with daratumumab was analyzed again after a median duration of follow -up of 14.7 months, median OS was 17.5 months (95% CI: 13.7 to NE). Forty-four percent of patients had died, and 6- and 12-month OS rates were 81.8 months and 64.7 months, respectively. Importantly, daratumumab demonstrated a highly favorable safety profile. Therapy was well tolerated with manageable side effects, and no subject discontinued treatment because of a drug-related treatment-emergent adverse event (TEAE) or an infusion-related reaction (IRR). The most common (≥20%) TEAEs included fatigue (39.6%), anemia (33.0%), nausea (29.2%), thrombocytopenia (25.5%), neutropenia (22.6%), back pain (21.7%), and cough (20.8%). The most common Grade 3 or 4 TEAEs (>10%) were anemia (23.6%), thrombocytopenia (18.9%), and neutropenia (12.3%). The most frequently reported serious TEAEs (≥3%) were general physical health deterioration (4.7%), pneumonia (3.8%), and hypercalcemia (3.8%). Infusion-related reactions (IRRs) occurred in 42.5% of subjects. The vast majority (>90%) of these IRRs were Grade 1 or 2 in severity and occurred during the first two infusions. The use of hematopoietic growth factors or transfusions was low. Overall, the data from the pivotal Study MMY2002 demonstrated that daratumumab monotherapy has a highly favorable benefit/risk profile in heavily pretreated patients with MM who otherwise have very limited treatment options.

## 3. Phase I/II Study GEN501

Results from Study GEN501 provide additional support for the efficacy and safety of daratumumab monotherapy in patients with heavily pretreated MM. The two-part trial GEN501 was the first study to establish a positive benefit/risk profile for daratumumab monotherapy in the treatment of patients with MM whose disease was relapsed or refractory to at least two prior lines of therapy and who did not have further established treatment options. The response to daratumumab 16 mg/kg in Study GEN501 was similar to that in Study MMY2002, with subjects having an ORR of 36%. Responses were also consistent across multiple patient subgroups and were rapid (<1 month) and durable. Survival results were highly favorable, with a median PFS of 5.6 months and a 6-month OS rate of 88% (median OS not reached). Daratumumab additionally showed compelling tolerability, though in a less heavily pretreated MM population compared with that in Study MMY2002.

## 4. Integrated Results

Analysis of the integrated efficacy and safety results for daratumumab monotherapy underscores the similarity between the results of Studies MMY2002 and GEN501. Integration of data from Study MMY2002 and Part 2 of Study GEN501 resulted in an ORR of 31% and a duration of response of 7.6 months. The overall median time to first response was slightly less than one month, corresponding with the first disease assessment. A remarkable depth of response (VGPR or better) of 11% was observed. Similarly, PFS (4.0 months; 95% CI: 3.0–5.6) and OS (19.9 months at 14.8-month follow-up; 95% CI: 15.1–NE) results were highly favorable, particularly when compared to those associated with other anti-MM treatments and RWE/historical controls. Treatment was well tolerated, with a favorable safety profile in subjects with heavily pretreated and refractory MM.

## 5. Daratumumab in Combination for Relapsed/Refractory Patients

In patients with at least one previous line of therapy, in 2017, daratumumab was also approved in combination with either bortezomib/dexamethasone (Vd) or lenalidomide/dexamethasone (Rd). This approval was based on two randomized phase III studies, that showed superior PFS and ORR using the MoAb-containing triplets daratumumab plus standard-of-care regimens (bortezomib–dexamethasone [CASTOR trial] [5] or lenalidomide–dexamethasone [POLLUX trial] [6].

## 6. CASTOR Trial 

In this phase 3 study, 498 patients with progression disease after at least one line of therapy, not refractory to any proteasome inhibitor or with peripheral neuropathy ≥grade 2, were randomized, in a 1:1 ratio, to receive daratumumab (experimental arm) in association to Vd or Vd alone. Patients received up to eight 21-days cycles of bortezomib and dexamethasone at the standard doses: 1.3 mg/m^2^ on days: 1, 4, 8, and 11, and 20 mg on days 1, 2, 4, 5, 8, 9, 10, and 11, respectively. Subjects in the experimental arm received 16 mg per kilogram of body weight intravenous daratumumab weekly during cycles 1 to 3, once every three weeks (on day 1) during cycles 4 to 8, and once every four weeks thereafter until the patient withdrew consent, the disease progressed, or unacceptable toxic effects developed. The primary endpoint was PFS as well as secondary endpoints, including the time to disease progression, the ORR, the proportion of patients who achieved very good partial response or better, the duration of response, the time to response, OS, and the time to subsequent anti-myeloma treatment. The median PFS was not reached in the daratumumab group versus 7.2 months in the control group (hazard ratio for disease progression or death with daratumumab vs. control, 0.39; 95% CI, 0.28 to 0.53; *p* < 0.001), which represented a 61.4% lower risk of progression or death in the daratumumab group than in the control group. In the daratumumab group, the ORR, the very good partial response, and the complete response or better were significantly higher (*p* < 0.001) than in the control group (82.9%, 59.2%, and 19.2% versus 63.2%, 29.1%, and 9.0%, respectively). The higher rate of thrombocytopenia, anemia, and neutropenia was observed in the daratumumab than in the control group 45.3%, 14.4%, and 12.8% versus 32.9%, 16.0%, and 4.2%, respectively. No significant differences in terms of peripheral sensory neuropathy, infection, or infestation Grade 3 or 4 were observed in the two groups. Forty-five percent of patients in the daratumumab arm experienced IRR, mostly Grade 1 or 2 (only 8.6% had Grade 3), that occurred in 98.2% of them during the first infusion. 

## 7. POLLUX Trial

The addition of daratumumab to lenalidomide and dexamethasone also significantly lengthened PFS among the same category of MM patients. Between June 2014 and July 2015, 569 relapsed or refractory MM patients were randomized in a 1:1 ratio to receive the standard therapy Rd with or without daratumumab. According to creatinine clearance, the lenalidomide dose was 25 or 10 mg for 21 days in 28-day cycles and dexamethasone 40 mg weekly reduced to 20 mg weekly for patients >70 years old. Intravenous daratumumab was administrated at a dose of 16 mg per kilogram of body weight once weekly during cycles 1 and 2, every two weeks during cycles 3 through 6, and continued every four weeks until disease progression or unacceptable toxicity. The first endpoint of the POLLUX study was PFS that, at 12 months, was 83.2% (95% CI, 78.3 to 87.2) vs. 60.1% (95% CI, 54.0 to 65.7) in the daratumumab and the control group, respectively. Among the second endpoints of the study, a significantly (*p* < 0.001) higher rate of overall response (92.9% vs. 76.4%), as well as a higher rate of complete response or better (43.1% vs. 19.2%), was obtained using the DRd treatment. The improved outcomes were associated with MRD negativity obtained in 22.4% of the patients treated with daratumumab versus 4.6% of those in the control group. Neutropenia, thrombocytopenia, and anemia were the most common adverse event of Grade 3 or 4 observed in the daratumumab group vs. the control group (51.9% vs. 37.0%; 12.7% vs. 13.5%; 12.4% vs. 19.6%). Forty-seven percent of patients in the daratumumab arm experienced IRR mostly Grade 1 or 2. In addition, Grade 3 or 4 of non-hematologic adverse events such as infection, diarrhea, fatigue, nausea, and dyspnea were slightly higher in the DRd group than in the Rd group. 

## 8. Daratumumab in Combination for New Diagnosis Patients

Therefore, the treatment with the most effective regimen in the frontline setting may provide the best approach to achieve deep and durable clinical responses. MoAbs, currently available for real-life clinical use in relapsed/refractory MM patients, seems to be almost ready to represent, in the near future, the backbone paradigms of both younger and elderly patients with a new diagnosis of MM (NDMM) as demonstrated by the study ALCYONE [7], MAIA [8], and CASSIOPEA [9].

## 9. ALCYONE Study

In 2018, daratumumab was the first MoAb approved as an upfront treatment for MM in combination with bortezomib/melphalan/prednisone (VMP) for transplant-ineligible patients based on randomized phase III data from the ALCYONE study, again showing a PFS benefit of the quadruplet regimen. In this study, 706 transplant-ineligible patients with NDMM were randomized 1:1 to receive either daratumumab with VMP (D-VMP) or VMP alone. The latter regime is one of the standard care for this category. Patients received oral melphalan and prednisone at 9 and 60 mg/m^2^, respectively on days 1, 2, 3, and 4, and bortezomib 1.3 mg/m^2^ on weeks 1, 2, 4, and 5 (twice a week during cycle 1 and weekly during cycle 3 through cycles 9). In the D-VMP arm, the MoAb was administrated intravenous at a dose of 16 mg per kilogram of body weight once weekly during cycles 1 and 2, every two weeks during cycles 3 through 6, and continued every four weeks until disease progression or unacceptable toxicity. The study met its primary endpoint at a median follow-up of 16.5 months, demonstrating a significant increase in the 18-month PFS benefit of 71.6% versus 50.2% (*p* < 0.001), favoring the daratumumab-containing quadruplet (ORR 90.9% vs. 73.9%, *p* < 0.001) with the rate of very good partial response or better significantly higher in the daratumumab group than in the control group (71.1% vs. 49.7%, *p* < 0.001), as was the rate of complete response or better (42.6% vs. 24.4%, *p* < 0.001). Median PFS at 1-year additional follow-up (median, 27.8 months) was not reached in the D-VMP arm compared with 19.0 months in the VMP arm, although the data have not matured enough to report overall survival, and follow-up for long-term survival is ongoing. Importantly, 22.3% of the patients in the D-VMP arm were MRD-negative compared with 6.2% in the VMP-alone arm. Although patients with high-risk cytogenetics favored the D-VMP arm, the hazard ratio was not significant. Not surprisingly, myelosuppression was the most common Grade 3 or 4 adverse event, which was similar between arms. The usual chemotherapy-related toxic effects were not increased by the addition of daratumumab as demonstrated by the neutropenia (in 39.9% of the patients in the daratumumab group and 38.7% of those in the control group), thrombocytopenia (in 34.4% and 37.6%, respectively), and anemia (in 15.9% and 19.8%, respectively) that were reported in this study. Nevertheless, there was an increased rate of Grade 3 or greater infections with D-VMP (23% vs. 15%) and the most common infection was pneumonia. Daratumumab-related infusion reactions (mostly of Grade 1 or 2) occurred in 27.7% of the patients, with the majority during the first infusion. 

## 10. MAIA Study

Given the encouraging responses improved by the addition of daratumumab to VMP, other studies are examining frontline combinations as well as the phase III MAIA study presented first as a late-breaking abstract during the 2018 ASH Annual Meeting and then published by Facon et al. in N. Eng J Med in 2019. In this randomized, open-label, phase 3 trial, 737 patients—368 to the daratumumab group and 369 to the control group—were randomly assigned to receive oral lenalidomide (25 mg on days 1 through 21) and oral dexamethasone (40 mg on days 1, 8, 15, and 22) until disease progression or unacceptable toxic effects with or without intravenous daratumumab at a dose of 16 mg per kilogram of body weight once weekly during cycles 1 and 2, every two weeks during cycles 3 through 6, and every four weeks thereafter. The primary endpoint was PFS and the secondary efficacy endpoints included the time to progression; overall response (including partial response, very good partial response, complete response, stringent complete response, very good partial response or better); and negative status for MRD, OS, time to response, duration of response, and safety. After a median follow-up of 28 months, the percentage of patients with an overall response was 92.9% in the daratumumab group and 81.3% in the control group (*p* < 0.001). In particular, the percentage of patients with a complete response or very good partial response was significantly higher in the daratumumab group than in the control group 47.6% vs. 24.9% and 79.3% vs. 53.1%, respectively (*p* < 0.001 for both comparisons). The median PFS was not reached in the daratumumab group and was 31.9 months (95% CI, 28.9 to not-reached) in the control group, and its benefit was maintained among patients 75 years of age or older (hazard ratio, 0.63; 95% CI, 0.44 to 0.92). At 30 months of observation, 70.6% (95% confidence interval (CI), 65.0 to 75.4), and 55.6% (95% CI, 49.5 to 61.3) of patients were alive in response in the daratumumab group and the control group, respectively. With regard to MRD negativity in the daratumumab group, the percentage of patients was significantly higher (24.2% vs. 7.3%, *p* < 0.001). All the patients who were negative for MRD had a complete response or better. The median OS was not reached in either group, and follow-up for long-term survival is ongoing. Neutropenia, anemia, lymphopenia, and pneumonia were the most common adverse event of Grade 3 or 4 observed in the daratumumab group versus the control group (50% vs. 35.3%; 11.8% vs. 19.7%; 15.1% vs. 10.7%; 13.7% vs. 7.9%). 

## 11. CASSIOPEA Study

In the CASSIOPEIA study, 1085 transplant-eligible patients with NDMM were randomized 1:1 to receive either intravenous daratumumab with bortezomib, thalidomide, and dexamethasone (VTd) or VTd alone. In this phase III study, patients received up to four 28-day cycles as induction and two 28-day cycles as consolidation therapy, oral thalidomide (100 mg/day), bortezomib (1.3 mg/m² on days 1, 4, 8 and 11 of each cycle) and dexamethasone (40 mg on days 1, 2, 8, 9, 15, 16, 22, and 23 of cycles 1 and 2, on days 1 and 2 of cycles 3 and 4, and 20 mg on days 8, 9, 15, and 16 of cycles 3 and 4 of induction treatment, and on days 1, 2, 8, 9, 15, and 16 of both consolidation cycles). In addition, 543/1085 in the daratumumab group received MoAb at a dose of 16 mg/kg weekly in induction cycles 1 and 2 and then once every two weeks during induction cycles 3 and 4 and consolidation. At the end of induction therapy, all patients received cyclophosphamide and granulocyte colony-stimulating factor as stem-cell mobilization therapy and then proceed to autologous stem-cell transplantation after intravenous melphalan of 200 mg/m². Consolidation therapy started at least 30 days from the transplant and haematopoietic reconstitution. At 100 days, post-transplant patients, at least in PR, underwent a second randomization to observation or maintenance therapy with daratumumab (16 mg/kg) every 8 weeks until disease progression or for a maximum of 2 years. The study was designed to evaluate, as the primary endpoint, the proportion of patients who achieved an sCR after consolidation. The proportion of patients who were MRD negative and achieved a complete response or better after consolidation, PFS, and OS from first randomization were evaluated as second endpoints. A significant (*p* < 0.0001) higher proportion of patients who achieved an sCR and a complete response or better was observed in the daratumumab group versus control group: 39% vs. 26%, 29% vs. 20%, respectively, as well as a very good partial response or better: 83% vs. 78% (*p* = 0.024). Importantly, the negative status for MRD following consolidation, assessed by multiparametric flow cytometry, or by next-generation sequencing, was 64% vs. 44% (*p* < 0.0001) and 57% vs. 37% (*p* < 0.0001), in the D-VTd arm versus VTd-alone, respectively. The median PFS and OS were not reached in either group. At 18 months of observation, the probability of PFS was 93% (95% CI 90–95) and 85% (95% CI 81–88) with 14 and 32 patients death in the D-VTd and VTd group, respectively. With regard of Grade 3 and 4 adverse event neutropenia, lymphopenia and stomatitis were the most common observed in the daratumumab group versus the control group (28% vs. 15%, 17% vs. 10%, 13% vs. 16%). Sixty-five percent vs. 57% experienced infections of any grade, but infections Grade 3 or 4 were similar (22% vs. 20%). The proportion of patients proceeding to autologous stem-cell transplantation did not differ between groups nor did the proportion of patients achieving haematopoietic reconstitution. Median numbers of CD34+ cells collected and the median numbers of cells transplanted were 6.3 × 10⁶ vs. 8.9 × 10⁶ per kg, and 3.3 × 10⁶ vs. 4.3 × 10⁶ per kg in the D-VTd group and the VTd group, respectively. Plerixafor was used during stem-cell mobilization in 22% of patients in the D-VTd group and 8% in the VTd group. However, there is an unmet need to evaluate other daratumumab-based combination regimens due to the consideration that some regimen, like VMP, is not commonly used in all countries. In the LYRA phase II study [10], for example, daratumumab was associated to bortezomib, cyclophosphamide, and dexamethasone (D-VCd) to treat NDMM and relapsed MM. Overall, D-VCd was well tolerated, the ≥VGPR rate after 4 cycles was 44% and the 1-year PFS rate was 87%. These data also warrant investigation of D-VCd efficacy in a controlled study.

## 12. New Daratumumab Combination for Relapsed/Refractory Patients

Daratumumab in combination with other agents has been investigated in RRMM patients. In a phase I study, 85 RRMM patients, after 1 to 3 prior lines of therapy, carfilzomib- and daratumumab-naive were treated with D-Kd (daratumumab, carfilzomib, and dexamethasone). Using this combination, 84% of patients obtained an ORR with 13% and 47% in sCR/CR and VGPR, respectively [11]. At 12 months, the PFS rate was 74% and the median PFS was not reached. The most common adverse events were similar to those reported in individual therapies. The association of daratumumab plus pomalidomide and dexamethasone (D-Pd) was recently approved in the U.S. by the U.S. Food and Drug Administration (FDA) in the RRMM patients based on the results of the EQUULEUS study [12]. In this phase Ib study, 103 patients were treated with D-Pd, 60% of them obtained an ORR (25% VGPR and 17% CR or better) with 29% MRD negativity. The 24-month PFS was 30% and the OS was 52%. As for the adverse events, these resulted similarly to Pd except for the higher incidence of neutropenia. Mostly due to progression disease, 82% of patients discontinued the treatment after a median follow up of 24.7 months. The association D-Kd and D-Pd are now under evaluation in ongoing phase III studies. In addition, in a phase Ib study, daratumumab was combined with KRd (D-KRd) in NDMM transplant eligible and ineligible patients [13]. This quadruplet combination resulted in 100% of ORR (57% sCR/CR and 33% VGPR), 95% PFS, and 100% OS at 12-months without a difference in terms of CD34+ cell collection yields when compared to patients treated with KRd. With regard to Grade 3 and 4 adverse events, pulmonary embolism was the most common (14%), and 27% of patients experienced IRRs (Grade 1 or 2). 

## 13. Toxicities

The main toxicity consists of infusion-related reactions (IRRs); however, treatment discontinuation due to IRRs is uncommon. In addition, the CD38 expression of the airway muscle cells partially explains the main symptoms that involve the respiratory tract, with throat irritation, cough, and dyspnea. In clinical practice, to prevent severe respiratory IRRs, standard premedication of MoAbs with steroids, antihistamines, and antipyretics administration is recommended. Based on the infusion time and incidence of IRRs associated with daratumumab accelerated infusion rates [14] or a split dose over two days, for cycle 1, day 1 [10,13], have been suggested. More interestingly is the subcutaneous delivery of the mix-and-deliver (MD) formulation of daratumumab in combination with the recombinant human hyaluronidase PH20 enzyme (rHuPH20) DARA-MD as demonstrated with the PAVO [15] study. In this study using the DARA-MD (1800 mg), the PK concentrations resulted similar to or greater than daratumumab administrated at 16 mg/kg IV infusion. The deep and durable responses were consistent with IV daratumumab in a similar patient population as well as the safety profile and the rate of IRRs. 

## 14. Isatuximab in Monotherapy for Relapse/Refractory Patients

Isatuximab (SAR650984) is a novel IgG1-k anti CD38 MoAb [16]. Its clinical activity was initially tested in a phase II study where it was used as a single agent in patients with RRMM. In the cohort treated with the highest dose (≥10 mg/kg), the ORR was 24% to 29%, with four weeks of median time to first response and 25 weeks of median response duration [17]. Isatuximab (Isa)-related infusion reactions (mostly of Grade 1 or 2) occurred in 37/73 (51%) of the patients, with the majority during the first infusion, and led to treatment discontinuation in 2 patients. The single-agent activity of Isa and its favorable safety profile make it a promising candidate for use in combination therapies. Isatuximab was also tested in combination with carfilzomib [18], with Rd [19], and with Pd [20] in three-phase Ib trials. Using carfilzomib, isatuximab, and dexamethasone, eight patients reached a response: in 6 patients a PR and in 2 patients a VGPR. Concerning the adverse events all were Grade 1–3, no patients discontinued the treatment and the IRRS, observed in 6 patients, were Grade 1 or 2 and occurred during the first infusion. Isatuximab combined with Rd give an ORR of 56% and the responses were also achieved in lenalidomide-refractory patients (ORR 52%), with a median PFS of 8.5 months. The same ORR (56%) was obtained using isatuximab in association with pomalidomide and dexamethasone (Pd). These triplets appeared to be well tolerated, with the main adverse events being IRRs. These encouraging clinical data supported the initiation of a Phase III study aimed to evaluate the efficacy and safety of isatuximab 10 mg/kg once a week/every 2 weeks in combination with Pd (154 patients) compared with Pd (153 patients) alone for the treatment of patients with RRMM (ICARIA-MM [21]. The first endpoint of the ICARIA study was the PFS that, at 11.6 months of follow up, was 11.5 vs. 6.5 moths with IsaPd and Pd, respectively (HR 0.596 (95% CI 0.44–0.81), *p* = 0.001. The second endpoints were the ORR, the OS, time to progression, PFS in patients with high-risk cytogenetics (del[17p], t[4;14], t[14;16]), duration of response, safety, Isa PK in combination with pomalidomide, immunogenicity, and health-related quality of life (HRQoL). Across the two groups, the median prior lines of therapy, the estimated GFR, the number of patients refractory to lenalidomide and to PI, and patients with high-risk cytogenetics were balanced. A significant difference (*p* < 0.0001) were observed in terms of ORR (≥PR): 60.4% vs. 35.3%; VGPR rate or better: 31.8% vs. 8.5; and MRD negativity (NGS, 10-5): 5.2% vs. 0%, between the patients treated with IsaPd or Pd, respectively. The treatment-emergent adverse events Grade ≥3 resulted in 86.8% IsaPd vs. 70.5% Pd and the most common were infections (42.8% IsaPd and 30.2% Pd), neutropenia (84.9% IsaPd and 70.1% Pd), febrile neutropenia (11.8% IsaPd and 2.0% Pd). Due to AEs, 7.2% and 12.8% patients discontinued and 7.9% and 9.4% patients died in the IsaPd and Pd arm, respectively. Among the 38.2% of IRRs, 2.6% were Grade 3–4. Data, in terms of overall survival, are not mature yet after a median treatment duration of 41 weeks (IsaPd) vs. 24 weeks (Pd), but a trend to OS improvement in IsaPd was observed (HR 0.687; 95% CI 0.461–1.023) [22].

Promising preliminary results are now being evaluated using other anti-CD38 MoAb such as MOR202 [23] or chimeric antigen receptor T cells or CD38 drug conjugates, bispecific antibodies. In addition, based on the results obtained in MM patients, the efficacy and safety of anti-CD38 MoAb are now being evaluated in other plasma cell dyscrasias such as smoldering MM and in immunoglobulin light-chain amyloidosis [24]. In the future, to better improve the outcome of our patients, it will be very important to personalize treatment. To better choose among the different treatment options, it will be necessary to identify biomarkers able to predict response. We summarized the efficacy and toxicity, obtained in the different studies, in Table S1 and Table S2, respectively.

## Supplementary Materials

The following are available online at https://www.mdpi.com/2073-4409/8/12/1629/s1. Table S1: Efficacy of anti-CD38 antibodies.; Table S2: Toxicities of anti-CD38 antibodies.
